# Supplementary material for: Improved betulinic acid biosynthesis using synthetic yeast chromosome recombination and semi-automated rapid LC-MS screening
Source: Nat Commun. 2020 Feb 13;11:868. doi: 10.1038/s41467-020-14708-z (PMC7018806; doi:10.1038/s41467-020-14708-z)
Supplement: Supplementary file 1 — Supplementary Information [file 41467_2020_14708_MOESM1_ESM.pdf]

Supplementary material for the publication:

Improved betulinic acid biosynthesis using synthetic yeast chromosomes, multiplexed nanopore sequencing, and ultra-fast LC-MS.

Gowers, G-O.F.[1,3], Chee, S-M.[2,5], Bell, D.[2,5], Suckling, L.[2,5], Kern, M.[6], Tew, D.[6], McClymont, D.W.[2,5], and Ellis, T.*[1,3]

[1]Imperial College Centre for Synthetic Biology, Imperial College London, SW7 2AZ, UK.

[2]London Biofoundry, Imperial College London, SW7 2AZ, UK.

[3]Department of Bioengineering, Imperial College London, SW7 2AZ, UK.

[4]Structural and Synthetic Biology, Department of Infectious Disease, Imperial College London, SW7 2AZ, UK.

[5]SynbiCITE, Imperial College London, SW7 2AZ, UK

[6]GlaxoSmithKline, Stevenage, SG1 2NY, UK

*Corresponding author (Email: t.ellis@imperial.ac.uk).

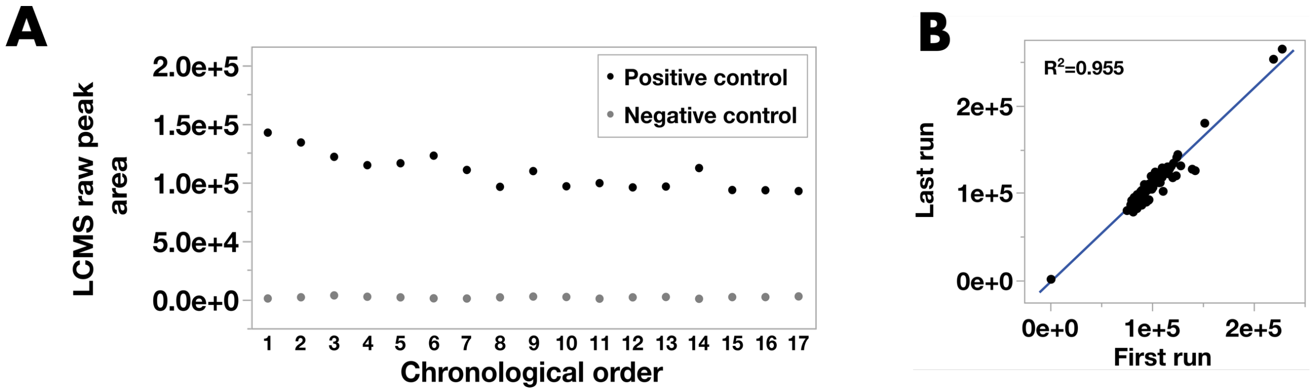

Supplementary Figure 1. A. A positive (1 mg/L betulinic acid in isopropanol) and negative sample (isopropanol only) was run at the start and end of every plate run (presented in chronological order) and at the start and end of each day to track analyte drift of the raw peak area of BA. A Z' value of 0.55 was calculated. A good assay expects to achieve a Z' value above 0.5. **B.** The same plate (with 48 SCRaMbLEd strains and 40 unSCRaMbLEd control strains) was run at the start and the end of the entire LC-MS screen period (2.5 days) to monitor sample changes during this time. Axis indicate LC-MS raw peak area values. Linear regression analysis (blue line) shows R^2=0.955. Source data are provided as a Source Data file.

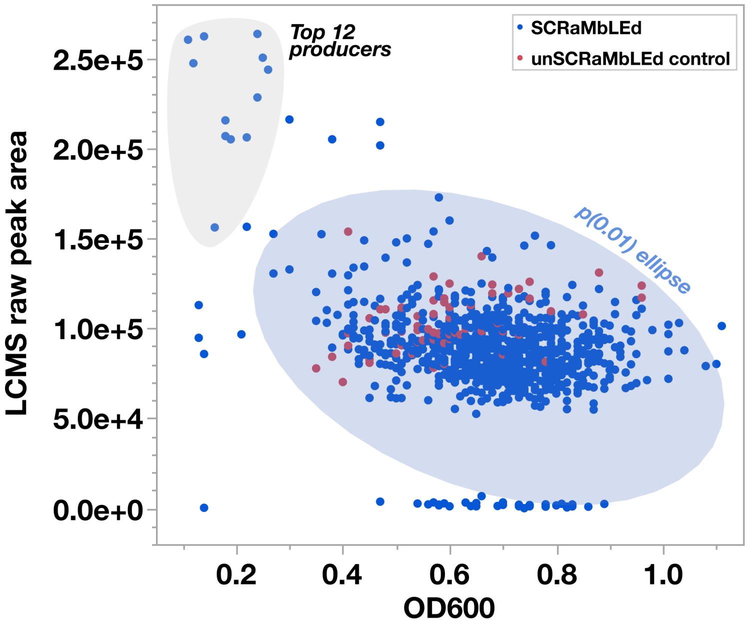

Supplementary Figure 2. Data from Fig 1C (main text). LC-MS raw peak area is plotted against OD_{600}, measured immediately before extraction, to identify the separate abilities of the SCRaMbLEd strains (blue) to grow and produce BA. A p(0.01) ellipse is shown (blue shading) where all control strains and 'unimproved' SCRaMbLE strains lie. Grey shading indicated the top12 strains from Fig 1C (main text). Source data are provided as a Source Data file.

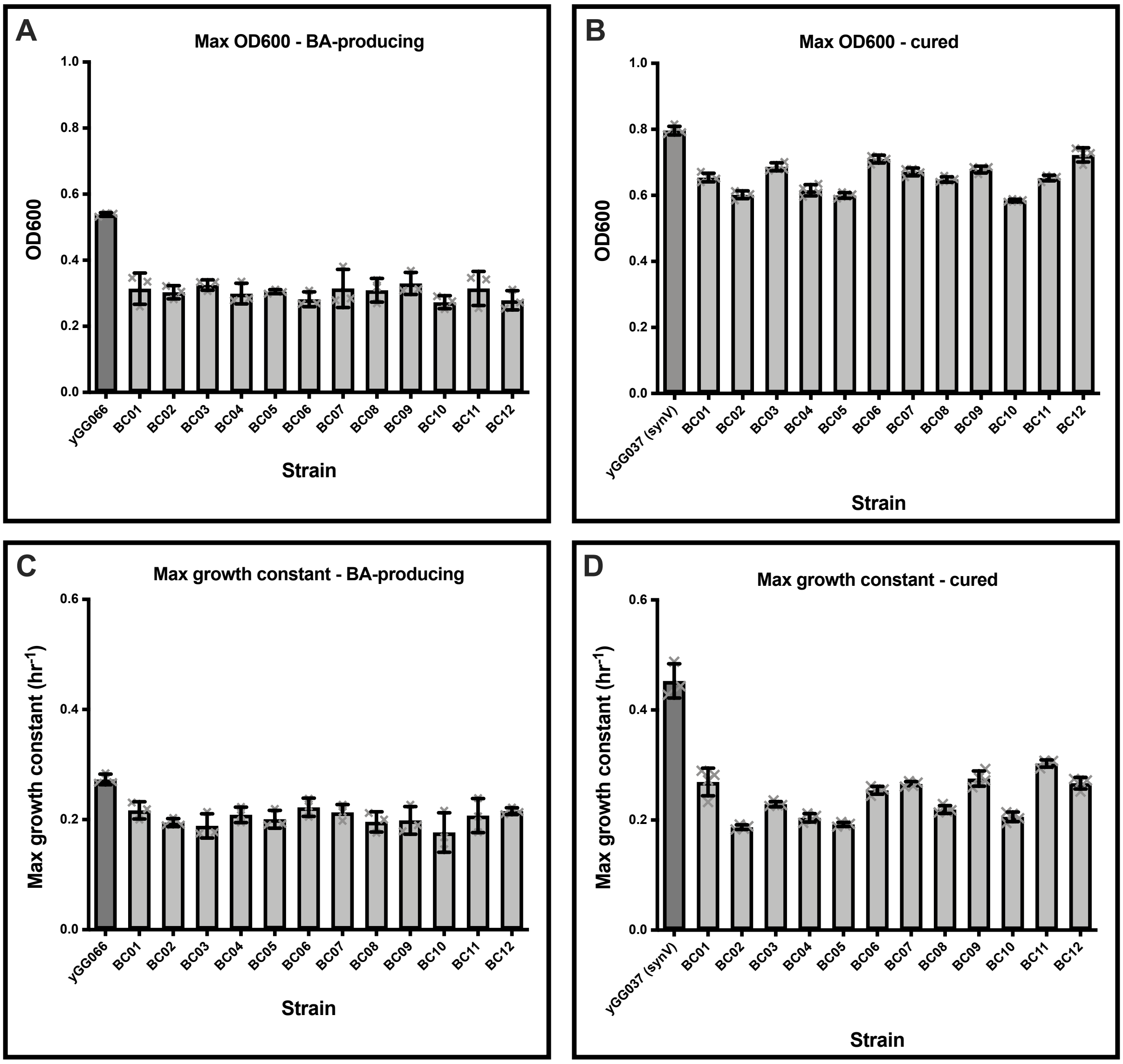

Supplementary Figure 3. Max OD_{600} (**A and B**) and max growth constants (**C and D**) for strains BC01-12. Strains with the BA plasmid are shown on the left **(A and C**, n=3 biologically independent samples**)** and strains cured of all plasmids is shown on the right **(B and D**, n=4, except yGG037 panel D (n=3), biologically independent samples**)**. Strains were picked into 500 µl URA- media (left) or 500 µl Synthetic Complete media (right) and grown overnight (30˚C, 700 rpm). Cultures were then used to inoculate 100 µl fresh media in a 96-well nunc plate. OD600 was measured every 15 minutes for 40 hours in a plate reader (Synergy HT) with 'medium' shaking in between every reading. Data are presented as mean values +/- standard deviation. Source data are provided as a Source Data file.

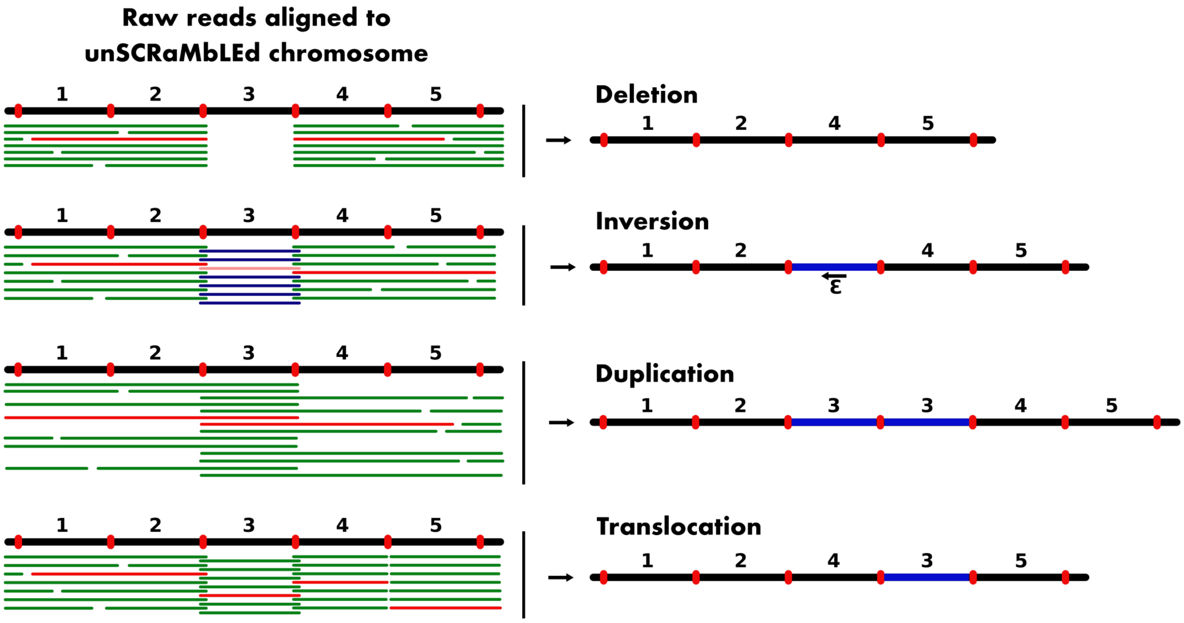

Supplementary Figure 4. Illustration to show how long read sequencing can resolve SCRaMbLE events. Lines indicate individual reads aligned to a pre-SCRaMbLE reference genome. LoxP sites (red circles) are distributed across the genome (black line) to divide the genome into inter-LoxP regions (numbered 1-5) that can be deleted, inverted, duplicated, and/or translocated. Green lines indicate reads aligned in a single direction. Purple lines indicate reads aligning in reverse orientation. In each case a single hypothetical read is shown as a red line that is contiguous in the top to bottom direction.

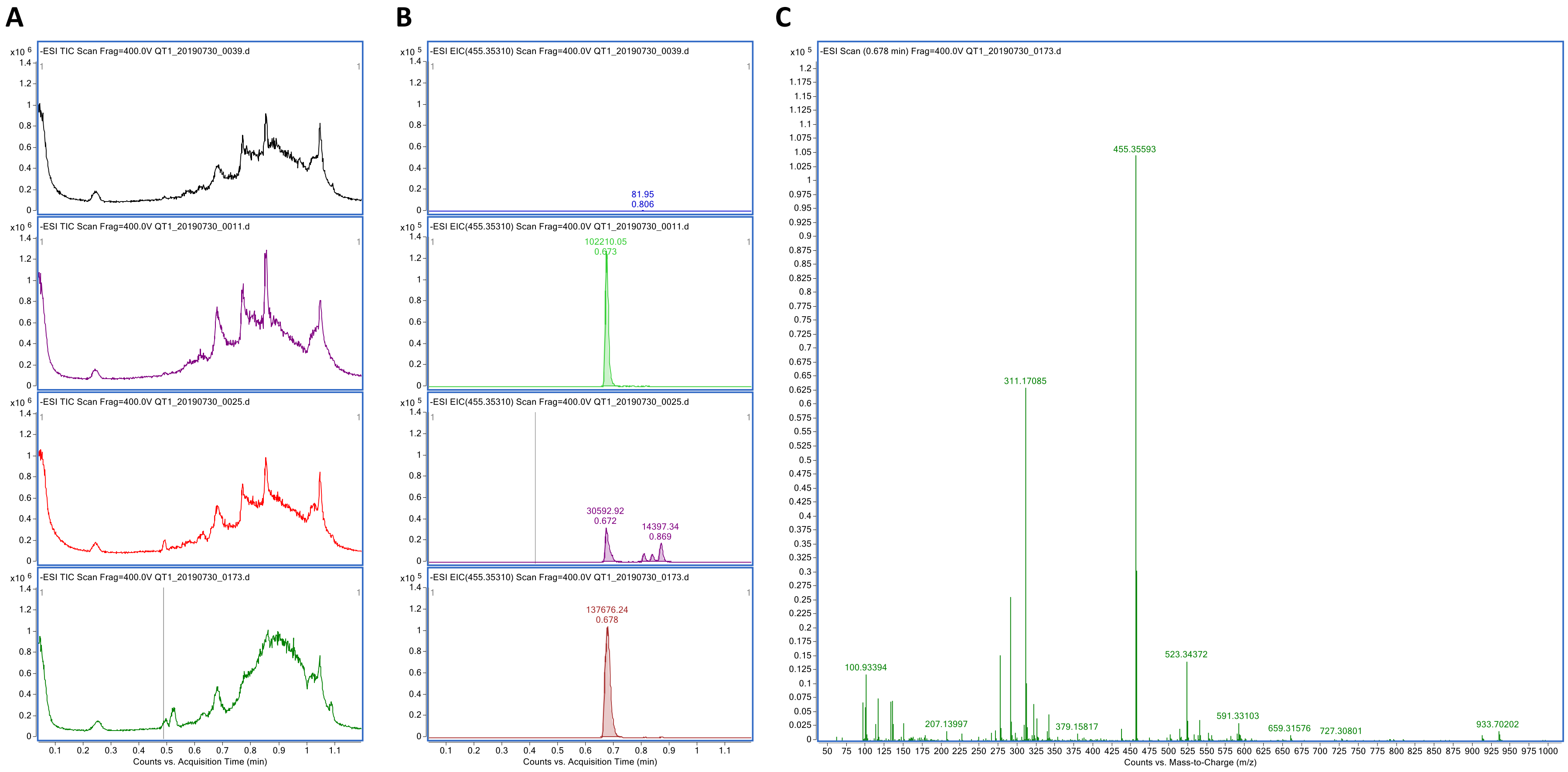

Supplementary Figure 5. (A) Total ion chromatograms and **(B)** extracted ion chromatograms of the target material in a negative control, a spiked negative control, and a low-and high-expressing culture (top to bottom). **(C)** A representative negative ion mass spectrum corresponding to betulinic acid (right).

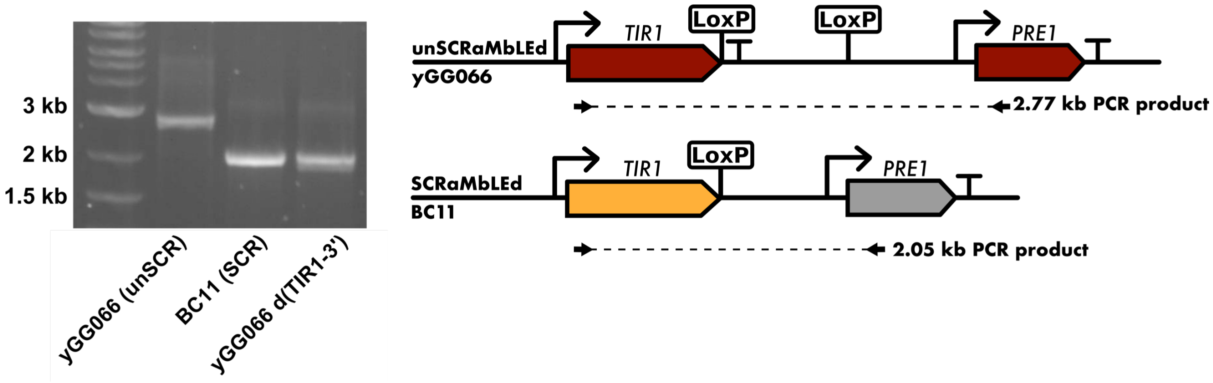

Supplementary Figure 6. PCR characterisation of yGG066 (pre-SCRaMbLE, right, top), BC11 (right, bottom), and yGG066 d(TIR1-3') (right, bottom) using primers GG154 (5'-GAACTGCGTTTGTATGCAACTG-3') and GG155 (5'-GGTAATTCGACTTTAGTACCCAAGTAGTC-3'). This PCR was performed independently twice with the same result in both cases.

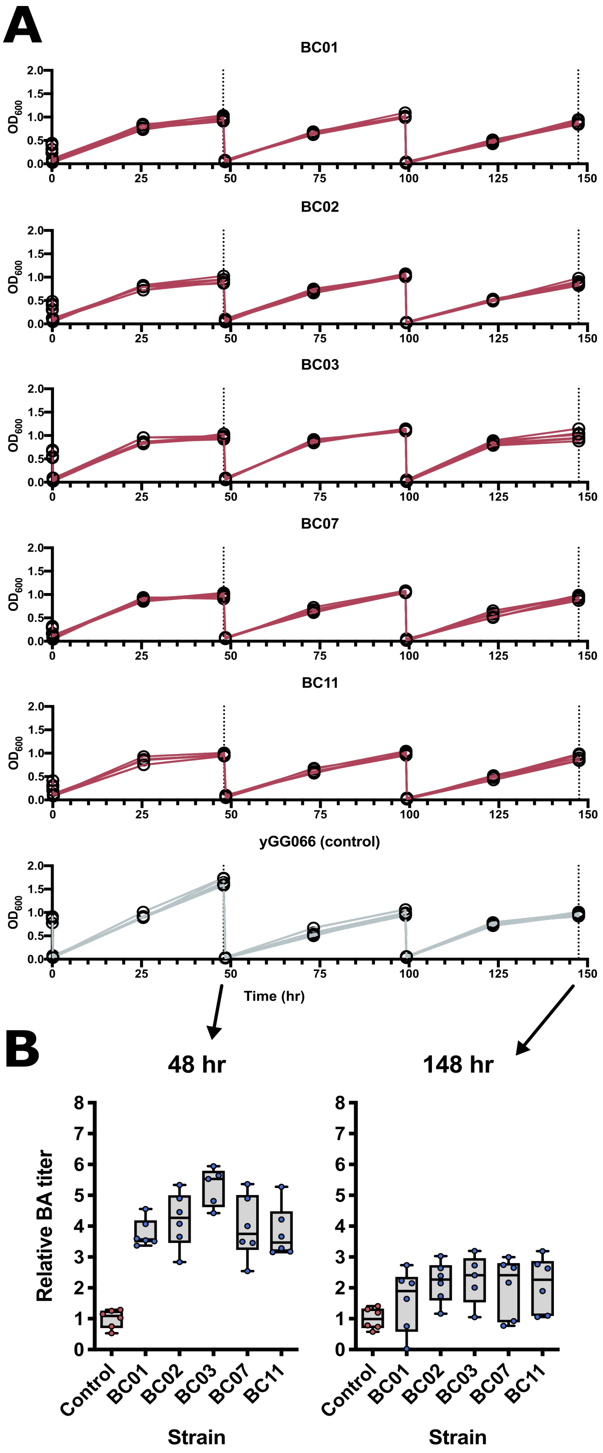

Supplementary Figure 7. Isolates of strains BC01, 2, 3, 7, and 11 and yGG066 were grown for three back dilutions over 3 days in 500 µl SDO URA- media shaking at 30˚C. **(A)** OD600 was measured and cultures were diluted back to OD600 0.1 every 48 hr. **(B)** Cultures were extracted for BA production at 48hr (after 1 growth cycle, left) and 148 hr (after 3 growth cycles, right). Data are presented as a box plot centred around the mean with bounds between the 25th and 75th percentile. Whiskers represent minima and maxima. All samples n=6, except BC03 which was n=5. The majority of isolates from each culture maintain improved BA titre. One or two isolates exhibit lowered BA production to pre-SCRaMbLE levels and only a single isolate of a single strain lost the ability to produce BA. Source data are provided as a Source Data file.

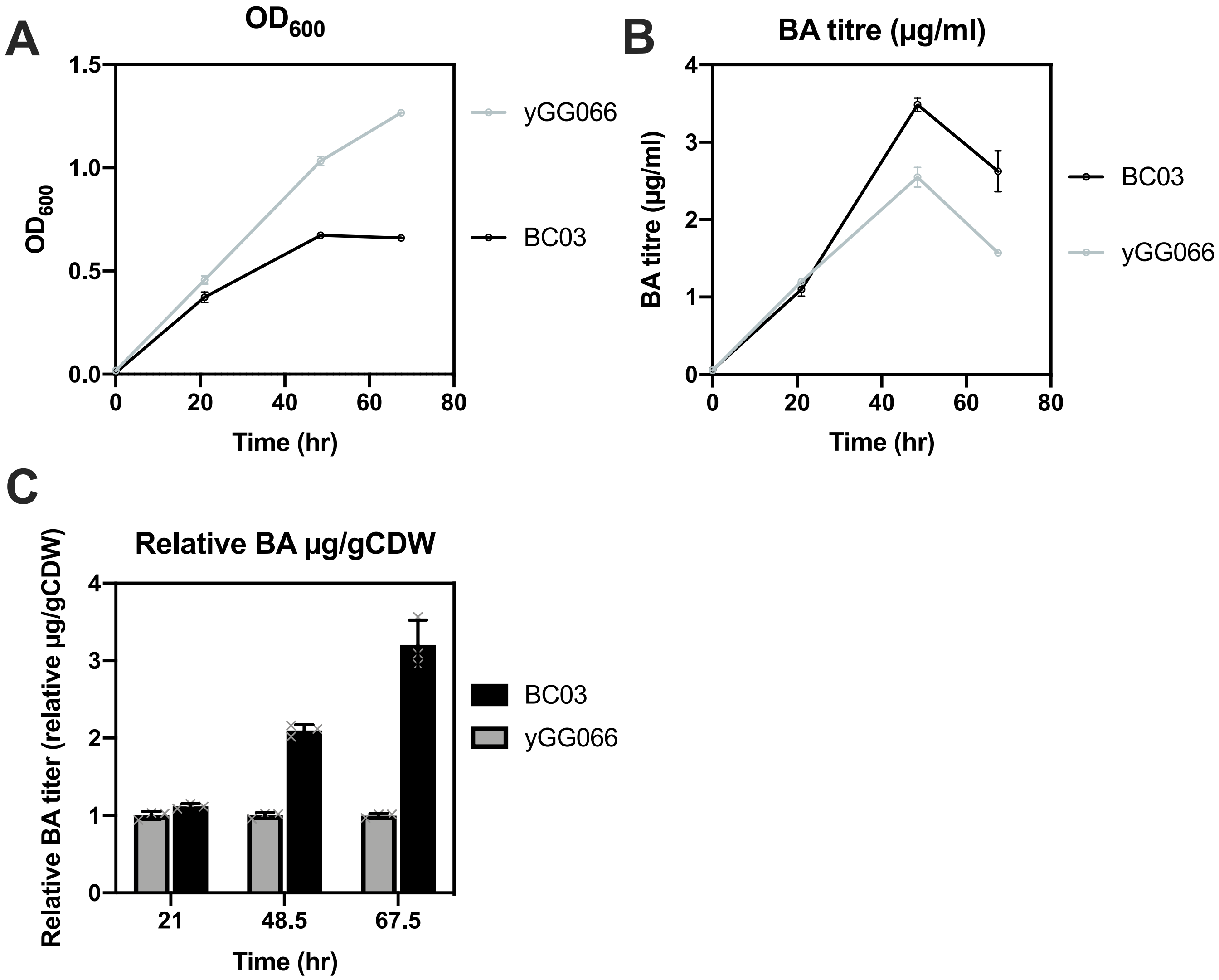

Supplementary Figure 8. Flask based cultures of yGG066 (pre-SCRaMbLE control) and BC03 (highest performer of this study). Cultures were inoculated into 250 ml baffled flasks containing 50 ml SDO URA- media to OD600 0.01. Flasks were left shaking at 30˚C, 150rpm, for 72 hours with 500 µl of culture collected at 0, 21, 48.5, and 67.5 hrs. OD600 was recorded on a plate reader **(A)** at each timepoint prior to BA extraction for LCMS analysis **(B)** (n=3 biologically independent samples for A and B). A spiked standard of 1 µg/ml was run alongside each timepoint. Relative BA titre **(C)** was calculated by normalising data from B (BA titre) by OD600 (A) then normalising by the yGG066 control at each timepoint (n=3 biologically independent samples). Data are presented as mean values +/- standard deviation. Source data are provided as a Source Data file.
