# Supplementary material for: Improved betulinic acid biosynthesis using synthetic yeast chromosome recombination and semi-automated rapid LC-MS screening
Source: Nat Commun. 2020 Feb 13;11:868. doi: 10.1038/s41467-020-14708-z (PMC7018806; doi:10.1038/s41467-020-14708-z)
Supplement: Supplementary file 7 — Reporting Summary [file 41467_2020_14708_MOESM7_ESM.pdf]

nature research

Corresponding author(s): Tom Ellis

Last updated by author(s): Jan 21, 2020

Reporting Summary

Nature Research wishes to improve the reproducibility of the work that we publish. This form provides structure for consistency and transparency in reporting. For further information on Nature Research policies, see Authors & Referees and the Editorial Policy Checklist .

Statistics

For all statistical analyses, confirm that the following items are present in the figure legend, table legend, main text, or Methods section.

n/a	Confirmed	
☐	☒	The exact sample size (*n*) for each experimental group/condition, given as a discrete number and unit of measurement
☐	☒	A statement on whether measurements were taken from distinct samples or whether the same sample was measured repeatedly
☐	☒	The statistical test(s) used AND whether they are one- or two-sided<br>*Only common tests should be described solely by name; describe more complex techniques in the Methods section.*
☐	☒	A description of all covariates tested
☐	☒	A description of any assumptions or corrections, such as tests of normality and adjustment for multiple comparisons
☐	☒	A full description of the statistical parameters including central tendency (e.g. means) or other basic estimates (e.g. regression coefficient) AND variation (e.g. standard deviation) or associated estimates of uncertainty (e.g. confidence intervals)
☐	☒	For null hypothesis testing, the test statistic (e.g. *F*, *t*, *r*) with confidence intervals, effect sizes, degrees of freedom and *P* value noted<br>*Give P values as exact values whenever suitable.*
☒	☐	For Bayesian analysis, information on the choice of priors and Markov chain Monte Carlo settings
☒	☐	For hierarchical and complex designs, identification of the appropriate level for tests and full reporting of outcomes
☒	☐	Estimates of effect sizes (e.g. Cohen's *d*, Pearson's *r*), indicating how they were calculated

Our web collection on statistics for biologists contains articles on many of the points above.

Software and code

Policy information about availability of computer code

Data collection	Nanopore sequencing data was collected using Oxford Nanopore Minknow software version 18.12.4. PIXL imaging software (preinstalled on the PIXL robot, Singer Instruments) was used during colony picking (version 2.18.0920.1/2.12)
Data analysis	Reads were demultiplexed using Porechop (https://github.com/rrwick/Porechop) and aligned to pre-SCRaMbLE synV sequence using LASTAL (-l100 flag) (http://last.cbrc.jp/). Output files were converted to indexed BAM files using maf-convert (http://last.cbrc.jp/) and samtools (http://samtools.sourceforge.net/). Alignments were searched for SCRaMbLE events using Tablet (v1.19.05.28) using a synV.gff file to identify recombination events aligning with LoxPsym boundaries.

For manuscripts utilizing custom algorithms or software that are central to the research but not yet described in published literature, software must be made available to editors/reviewers. We strongly encourage code deposition in a community repository (e.g. GitHub). See the Nature Research guidelines for submitting code & software for further information.

Data

Policy information about availability of data

All manuscripts must include a data availability statement. This statement should provide the following information, where applicable:

- Accession codes, unique identifiers, or web links for publicly available datasets
- A list of figures that have associated raw data
- A description of any restrictions on data availability

Sequencing data is deposited in the SRA (SRR9988267). All data in figures has been made available in the supplementary source data file indicated in the Data Availability Statement)

Field-specific reporting

Please select the one below that is the best fit for your research. If you are not sure, read the appropriate sections before making your selection.

- [x] Life sciences
- [ ] Behavioural & social sciences
- [ ] Ecological, evolutionary & environmental sciences

For a reference copy of the document with all sections, see nature.com/documents/nr-reporting-summary-flat.pdf

Life sciences study design

All studies must disclose on these points even when the disclosure is negative.

Sample size	A sample size of 1000 strains was chosen as a benchmark for throughput an order of magnitude higher than a single 96-well plate.
Data exclusions	964 SCRaMbLE strains were picked for Fig 1C. 50 data points were excluded during analysis as measured OD600 value did not exceed our pre-established threshold of OD600 0.1 (indicating insufficient growth or a failed picking event).
Replication	To ensure repeatability as many biological and technical replications were run as possible for each experiment, this is indicated in the figure legends where appropriate. For all the data shown results were similar and consistent across all repeats.
Randomization	When screening 1,000 strains the strains were picked at random from agar plates without human input. This ensured no human bias was introduced when selecting strains for screening. Selecting strains was automatically done by the PIXL robot software (Singer Instruments, version 2.18.0920.1/2.13).
Blinding	Investigators were blinded during strain picking as this process was automated by robotic equipment (see above 'Randomization' section). After this point blinding was not appropriate as the authors actively sought out high performing strains to characterize in depth.

Reporting for specific materials, systems and methods

We require information from authors about some types of materials, experimental systems and methods used in many studies. Here, indicate whether each material, system or method listed is relevant to your study. If you are not sure if a list item applies to your research, read the appropriate section before selecting a response.

Materials & experimental systems

n/a	Involved in the study	
☒	☐	Antibodies
☒	☐	Eukaryotic cell lines
☒	☐	Palaeontology
☒	☐	Animals and other organisms
☒	☐	Human research participants
☒	☐	Clinical data

Methods

n/a	Involved in the study	
☒	☐	ChIP-seq
☒	☐	Flow cytometry
☒	☐	MRI-based neuroimaging
