# Supplementary material for: Improved betulinic acid biosynthesis using synthetic yeast chromosome recombination and semi-automated rapid LC-MS screening
Source: Nat Commun. 2020 Feb 13;11:868. doi: 10.1038/s41467-020-14708-z (PMC7018806; doi:10.1038/s41467-020-14708-z)
Supplement: Supplementary file 8 — Description of Additional Supplementary Files [file 41467_2020_14708_MOESM8_ESM.pdf]

Title: Supplementary Data 1, LC gradient conditions
Description: Summary of the LC buffer gradient used solvent A, 50% of methanol in 0.1% (v/v) formic acid in water; solvent B, 0.1% (v/v) formic acid in acetonitrile

Title: Supplementary Data 2, Plasmid sequences used in this study
Description: pGG127 was digested with Not1 and integrated into the HO locus of BY4741 yeast. pGG052 was transformed into this strain to generate yeast strain yGG066 used in this study.

Title: Supplementary Data 3, Gene sequences
Description. Gene sequences for non-standard YTK parts. Each sequence was integrated into pYTK001 for further construction (see main text).

Title: Supplementary Data 4, Summary of observed SCRaMbLE events
Description: Summary table indicating the type of and size of significant SCRaMbLE events that were observed SCRaMbLE strains (BC01-12). No sizable SCRaMbLE event was observed for BC05, 06, 12.
